# Supplementary material for: Distinct tissue niches direct lung immunopathology via CCL18 and CCL21 in severe COVID-19
Source: Nat Commun. 2023 Feb 11;14:791. doi: 10.1038/s41467-023-36333-2 (PMC9922044; doi:10.1038/s41467-023-36333-2)
Supplement: Supplementary file 1 — Supplementary Information [file 41467_2023_36333_MOESM1_ESM.pdf]

Distinct tissue niches direct lung immunopathology via CCL18 and CCL21 in severe COVID-19

Supplementary Figure 1

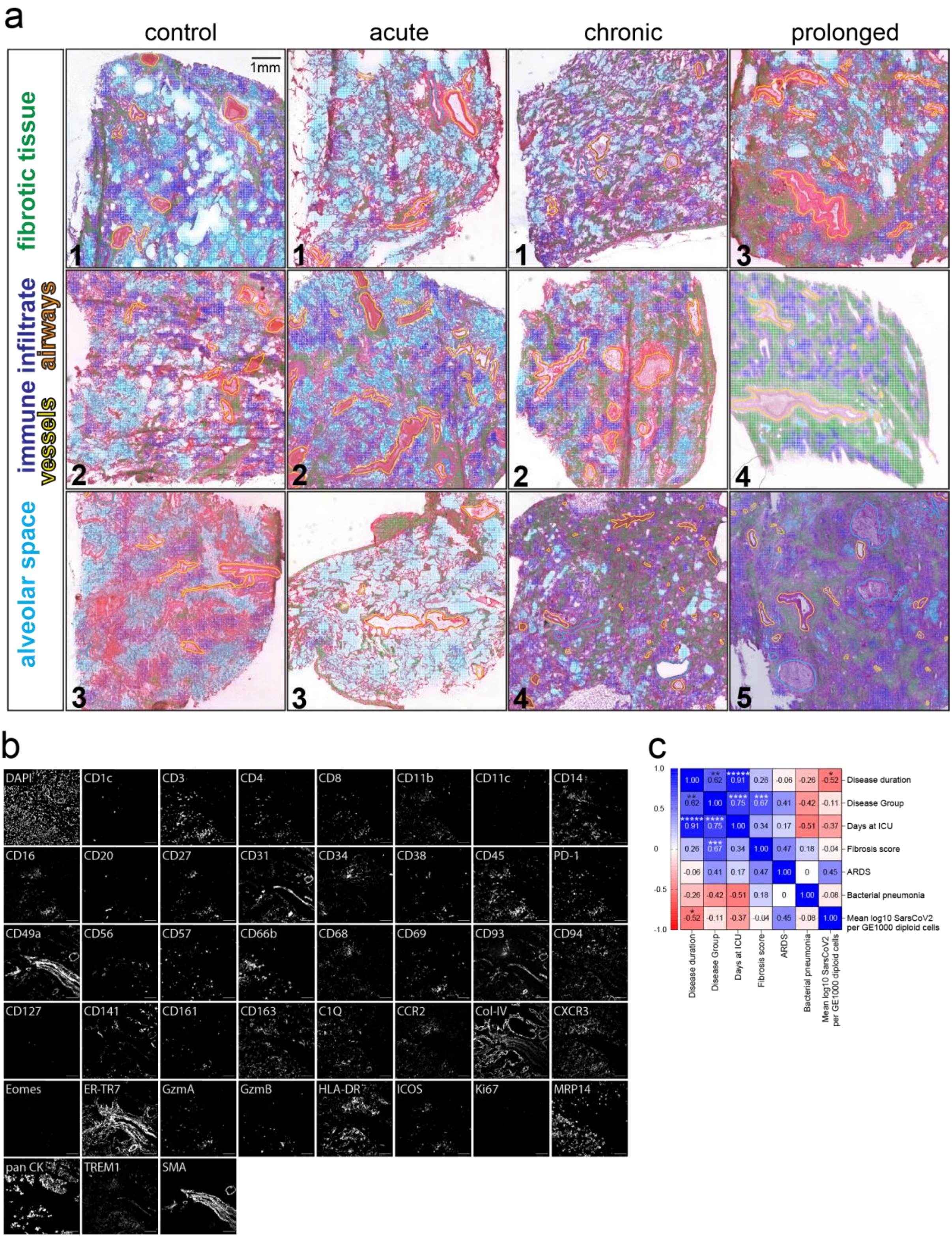

Supplementary Figure 1. **Overview of the histopathological and multiplex microscopy data of lung samples. a** HE staining of human lung tissue from non-COVID-related

pneumonia donors and COVID-19 cases was used by a blinded and trained pathologist to assess histopathological changes and to annotate recognizable lung structures to be used as landmarks for further analyses. Vessels are marked in yellow lines, airways in cyan lines, alveolar space in cyan areas, highly infiltrated areas in blue areas and fibrotic areas in green (n = 12 lung samples). Numbers represent donor IDs, as shown in Table 1. **b** Overview of 43-marker MELC panel in human lung. Each image depicts the same field of view (FOV) of 665 x 665 µm, sequentially stained with the depicted fluorescence-labelled antibodies. Images contain 2048 x 2048 pixels and are generated using an inverted wide-field fluorescence microscope with a 20x objective, a lateral resolution of 325 nm and an axial resolution above 5 µm. (Col.IV = collagen IV, GzmA = granzyme A, GzmB = granzyme B, pan CK = pancytokeratin). **c** Heat map display of the correlation matrix for relevant clinical parameters, donor stratification and fibrosis score, as evaluated by pathohistological examination of several lung sections from each donor based on Elastica van Gieson staining. See also Table 1. Pearson *r* values are shown in the plot with additional color code for positive (blue) or negative (red) correlations. Statistical significance (two-sided) is depicted with * for $p = 0.048$, ** for $p = 0.014$, *** for $p = 0.006$, **** for $p = 0.002$ and ***** for $p < 0.0001$.

Supplementary Figure 2

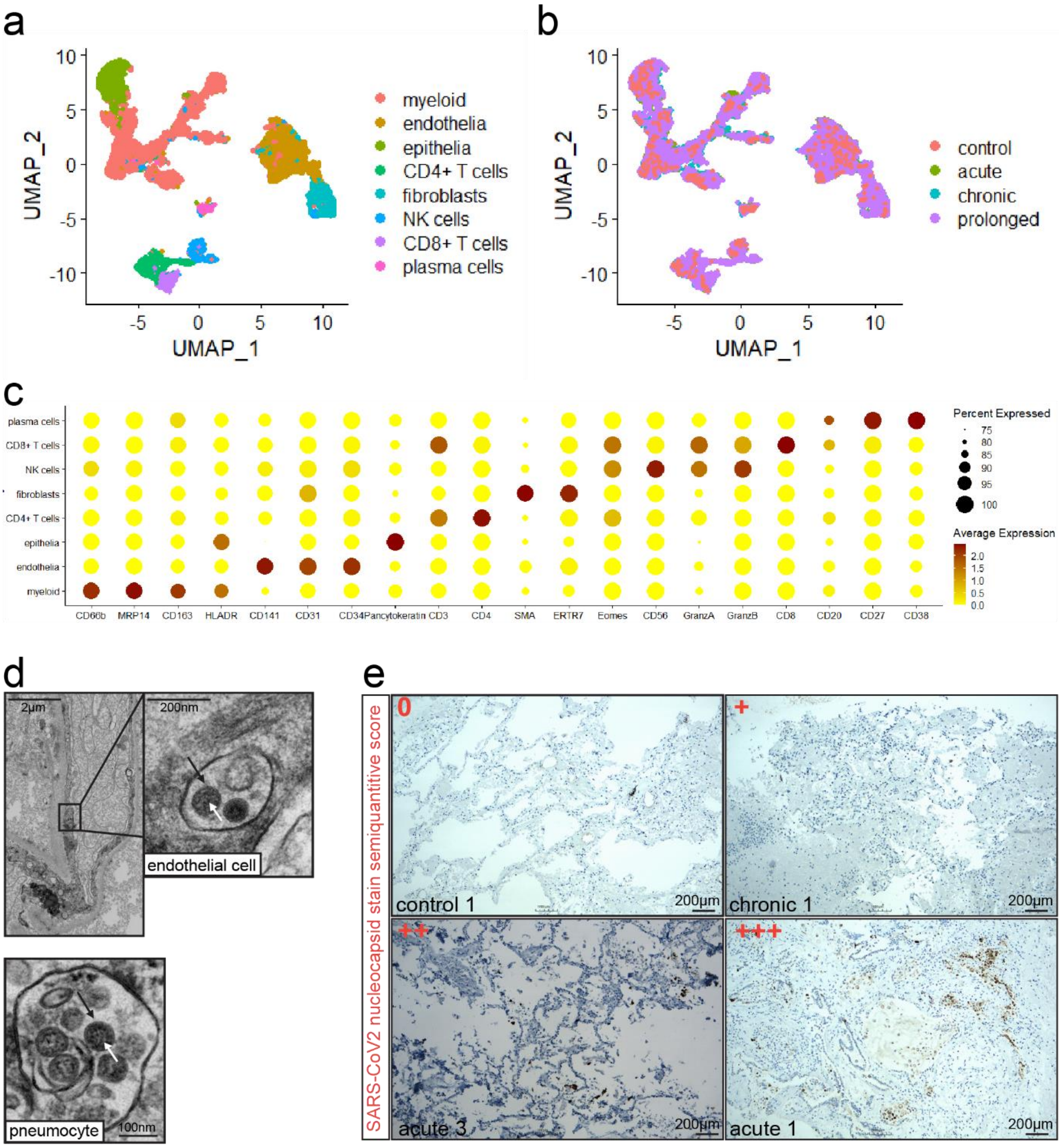

Supplementary Figure 2. **Analysis of cell composition within lung tissue and detection of coronavirus particles by microscopy. a, b** Dimensionality reduction and clustering analysis of segmented, single-cell multiplexed microscopy data of 32 fields of view (FOV) from 14 lung samples was performed (see material and methods) and visualized by Uniform Manifold Approximation and Projection (UMAP). Color code depicts the 8 annotated cell clusters (a), based on lineage-defining markers as shown in (c) or color code represents the disease group (b). **c** Dot plot showing the expression profile of the 8 cell clusters identified by multiplex

microscopy and defined in (a). **d** Ultrastructural images of an acute lung tissue sample showing two putative coronavirus particles within a membrane compartment in an endothelial cell. The coronavirus particles within pneumocytes can be unequivocally identified due to their larger number and, thus, as a collective clearly fulfil all morphologic criteria: faint but detectable surface projections (black arrow) and an electron dense, partly granular interior due to ribonucleoprotein (RNP, white arrow). In contrast, the two particles in the endothelial cell cannot unequivocally be identified as coronavirus particles, albeit they fulfill some morphologic criteria (surface projections; black arrow, possible RNP; white arrow), as we only detected these two examples in endothelial cells of six entirely digitized and analyzed ultrathin sections of autopsy lung of three different patients. **e** Representative images illustrating semiquantitative scoring of SARS-CoV-2 nucleocapsid immunohistochemical staining. See also Table 1.

Supplementary Figure 3

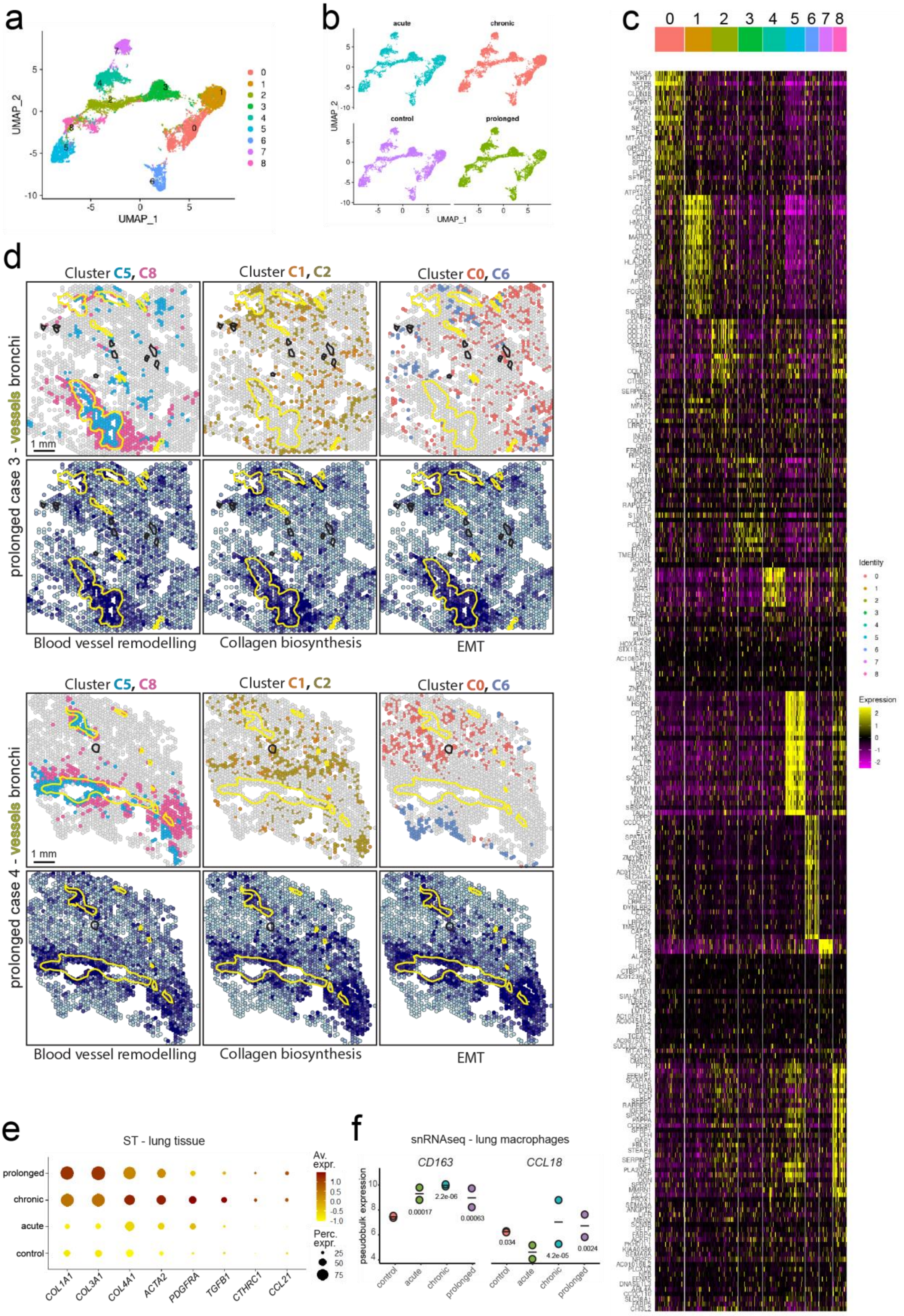

Supplementary Figure 3. **Identification of distinct lung tissue niches and their transcriptional signature.** Spatial transcriptomics (ST) feature spot-barcode expression matrices from 12 lung samples were filtered, normalized and integrated in R with Seurat (see methods). Uniform Manifold Approximation and Projection (UMAP) of ST data, where color code depicts the 8 identified spot clusters (**a**), or the disease group (**b**). **c** Heat map showing the top 25 differentially expressed genes for each cluster depicted in (a) (n = 12 lung tissue sections). **d** Color-coded tissue spots from two additional tissue sections of COVID-19 prolonged cases analyzed by ST, depict the spatial distribution of the relevant ST clusters, shown in Fig. 3b and (a - c), and the normalized enrichment scores (NES) of the gene set enrichment analysis (GSEA) from the Blood vessel remodelling (GOBP), Collagen biosynthesis and modifying enzymes (Reactome) and Epithelial to mesenchymal transition (Hallmark) pathways. Annotations for intermediate-to-large vessels (yellow) and airways (black) are shown as outlines of these tissue landmarks. **e** Dot plot depicting the NES and the percentage of expression of fibrosis-related genes in each disease group analyzed by ST (n = 12 lung tissue sections). **f** Dot plot of *CD163* and *CCL18* pseudobulk expression levels within the lung macrophage population for each disease group, analyzed by single nuclei RNA sequencing, as in [21]. Data (M ± SD) are analyzed by Wald test using DESeq2 on aggregated counts, where DF = 4 (n = 8 tissue samples).

Supplementary Figure 4

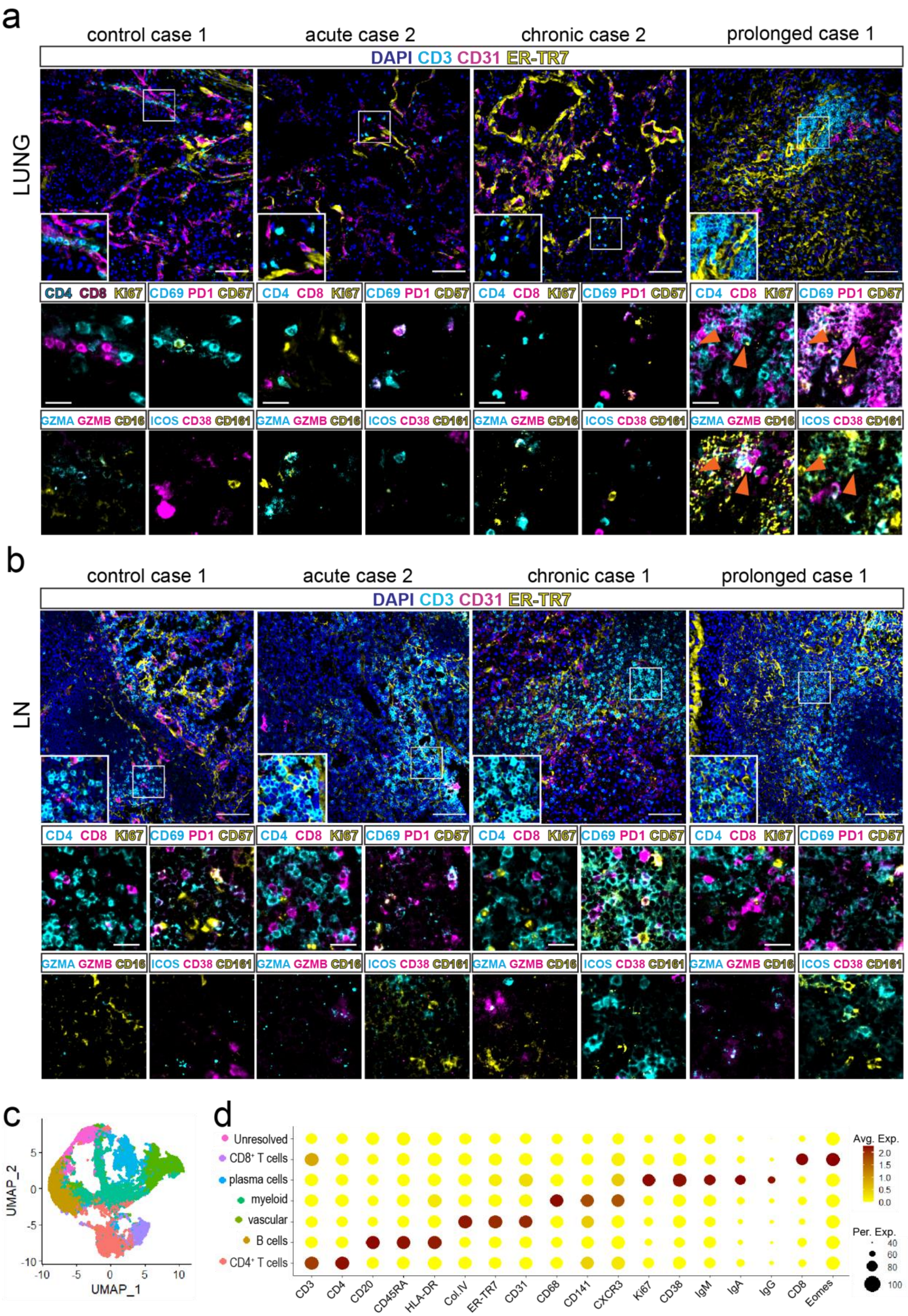

Supplementary Figure 4. **Exhausted T cells accumulate in fibrovascular niches of prolonged COVID-19 lungs, while T cells in the draining lymph nodes show a conventional activated profile. a** Representative multiplex microscopy images from each disease group showing nuclear staining DAPI (blue), the fibroblast marker ER-TR7 (yellow), the endothelial marker CD31 (magenta) and the T cell marker CD3 (cyan). White squares represent regions of interest (ROIs) shown as enlargements. For each lung area the identical ROI is depicted below in 4 overlays of different markers. Ki67 (yellow), CD4 (cyan) and CD8 (magenta); CD69 (cyan), PD1 (magenta) and CD57 (yellow); Granzyme A (cyan) and B (magenta) and CD16 (yellow); CD38 (magenta), CD161 (yellow) and ICOS (cyan) (n = 32). **b** The same arrays of overlays as in (a) are shown in the draining lymph nodes (n = 9). (a - b) Scale bars: 100 µm (complete field of view) or 25 µm (enlargements). **c** Uniform Manifold Approximation and Projection (UMAP) of segmented single-cell multiplex microscopy data from 7 lymph node samples. Color code depicts the annotated cell clusters, based on lineage-defining markers as shown in the dot plot (**d**), where the average and percentage of expression for each marker in each cluster is depicted.

Supplementary Figure 5

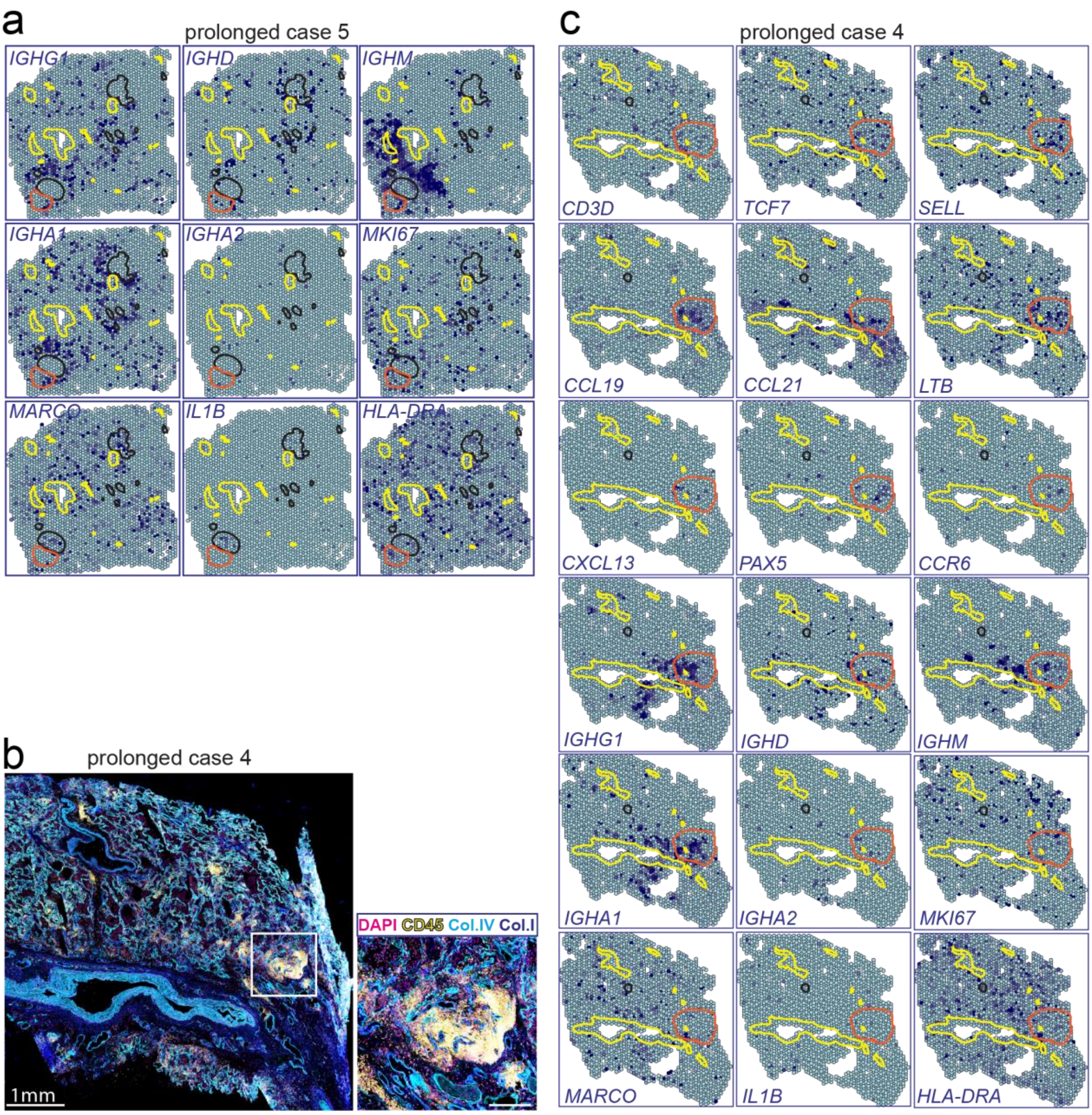

Supplementary Figure 5. **Spatial distribution of relevant transcripts. a** Color-coded tissue spots analyzed by spatial transcriptomics (ST) depict the tissue distribution of the normalized enrichment score (NES) for additional relevant transcripts within the same lung section from prolonged case 5 shown in Fig. 5a and b. **b** Immunofluorescence (IF) image depicting CD45 in yellow, DAPI in magenta, Collagen IV (Col.IV) in cyan and Collagen I (Col.I) in blue in an additional prolonged case. White square represents a region of interest (ROI), shown as an enlargement and depicting a dense immune cell aggregate. Scale bar: 250 µm. **c** The spatial distribution of transcripts reminiscent of tertiary lymphoid structure formation and additional relevant transcripts is shown in a color-coded fashion as NES in overlay with relevant tissue landmarks: intermediate-to-large vessels (yellow line), airway structures (black line) and lymphoid structure (orange line) (n = 2 lung samples). Capture areas (black squares) = 6 x 6 mm.

Supplementary Table 1

REAGENT or RESOURCE	SOURCE	IDENTIFIER	
**Antibodies**			**Dilution**
DAPI	Roche	Cat# 10236276001, N/A	1:5.000
Fibronectin	Thermo Fisher	Cat# PA5-29578; RRID:AB_2547054	1:200
Rabbit IgG-PE	Rockland	Cat# 711-708-127, RRID:AB_218957	1:200
CCR2-PE	Miltenyi Biotec	Cat# 130-118-338, RRID:AB_2751486	1:50
CD163-PE	Biolegend	Cat# 333605, RRID:AB_1134005	1:50
CD56-PE	Miltenyi Biotec	Cat# 130-098-137, RRID:AB_2661200	1:50
CCR8-APC	Biolegend	Cat# 360609, RRID:AB_2820017	1:50
CD1c-PE	Miltenyi Biotec	Cat# 130-113-864, RRID:AB_2726358	1:50
CD3-PE	Miltenyi Biotec	Cat# 130-113-139, RRID:AB_2725967	1:50
CD14-PE	Miltenyi Biotec	Cat# 130-113-709, RRID:AB_2726250	1:50
CCR7-PE	Miltenyi Biotec	Cat# 130-120-603, RRID:AB_2784046	1:50
Eomes-PE	Thermo Fisher	Cat# 14-4877-80, RRID:AB_2572881	1:50
CD45-PE	Miltenyi Biotec	Cat# 130-113-118, RRID:AB_2725946	1:50
CXCR3-PE	Miltenyi Biotec	Cat# 130-101-379, RRID:AB_2655734	1:10
PD1-PE	Miltenyi Biotec	Cat# 130-120-388, RRID:AB_2752074	1:50
CD16-PE	Miltenyi Biotec	Cat# 130-113-955, RRID:AB_2726428	1:50
CD93-PE	Miltenyi Biotec	Cat# 130-098-436, RRID:AB_2659615	1:10
CD4-PE	Miltenyi Biotec	Cat# 130-113-214, RRID:AB_2726025	1:50
Granzyme A-PE	Miltenyi Biotec	Cat# 130-123-973, RRID:AB_2889678	1:10
CD31-PE	R and D Systems	Cat# FAB3567P, RRID:AB_2279388	1:50
ICOS-PE	Miltenyi Biotec	Cat# 130-120-155, RRID:AB_2784102	1:50
TREM1-PE	Miltenyi Biotec	Cat# 130-101-033, RRID:AB_2657706	1:10
CD20-PE	Miltenyi Biotec	Cat# 130-113-374, RRID:AB_2726143	1:50
CD11b-PE	Miltenyi Biotec	Cat# 130-110-553, RRID:AB_2654665	1:50

CD8-PE	Miltenyi Biotec	Cat# 130-113-720, RRID:AB_2726261	1:50
CD68-PE	Miltenyi Biotec	Cat# 130-118-486, RRID:AB_2784270	1:50
CD127-PE	Miltenyi Biotec	Cat# 130-113-414, RRID:AB_2733759	1:50
CD11c-PE	Miltenyi Biotec	Cat# 130-113-580, RRID:AB_2726180	1:50
Granzyme B-PE	Miltenyi Biotec	Cat# 130-116-654, RRID:AB_2727639	1:50
CD69-PE	Miltenyi Biotec	Cat# 130-112-613, RRID:AB_2659065	1:50
CD94-PE	Miltenyi Biotec	Cat# 130-098-973, RRID:AB_2659624	1:10
CD141-PE	Miltenyi Biotec	Cat# 130-114-188, RRID:AB_2751233	1:50
CD27-PE	Miltenyi Biotec	Cat# 130-114-166, RRID:AB_2726471	1:50
CD38-PE	Miltenyi Biotec	Cat# 130-113-427, RRID:AB_2733813	1:50
CD57-PE	Miltenyi Biotec	Cat# 130-111-963, RRID:AB_2658747	1:50
CD161-PE	Miltenyi Biotec	Cat# 130-114-119, RRID:AB_2733771	1:50
HLA-DR,DP,DQ-PE	Miltenyi Biotec	Cat# 130-120-715, RRID:AB_2752176	1:50
CD34-PE	Miltenyi Biotec	Cat# 130-113-741, RRID:AB_2726281	1:50
CD66b-PE	Miltenyi Biotec	Cat# 130-122-966, RRID:AB_2811418	1:50
Pancytokeratin-PE	Arigo Biolaboratories	Cat# ARG56130	1:50
Ki67-FITC	Dako	F268	1:50
CD49a-PE	Miltenyi Biotec	Cat# 328304, RRID:AB_1236407	1:50
Collagen IV-FITC	Antibodies-Online	Cat# ABIN376119, RRID:AB_10763557	1:500
ER-TR7-PE	Thermo Fisher	Cat# MA1-40076, RRID:AB_1074409	1:200
SMA-FITC	Abcam	Cat# ab8211, RRID:AB_306359	1:100
CD45RA-PE	Miltenyi Biotec	Cat# 130-113-366, RRID:AB_2726136	1:50
C1q-FITC	DAKO	Cat# F0254, RRID:AB_2335713	1:50
MRP14-PE	Miltenyi Biotec	Cat# 130-114-516, RRID:AB_2726684	1:50
Collagen I-PE	Biolegend	Cat# 303126, RRID:AB_2563303	1:50
IgA-PE	Miltenyi Biotec	Cat# 130-114-002 RRID:AB_2733860	1:50
IgA2-PE	Miltenyi Biotec	Cat# 130-117-874 RRID:AB_2728061	1:50
IgM-PE	Miltenyi Biotec	Cat# 130-122-930 RRID:AB_2801972	1:50

IgG-PE	Miltenyi Biotec	Cat# 130-119-964 RRID:AB_2751950	1:50
CD45-AF647	Santa Cruz Biotechnology	Cat# sc-1178, RRID:AB_627074	1:50
CD3-AF647	Biolegend	Cat# 344825, RRID:AB_2563440	1:50
CD163-AF647	Biolegend	Cat# 326508, RRID:AB_893264	1:50
ER-TR7-AF546	Santa Cruz Biotechnology	Cat# sc-73355, RRID:AB_1122890	1:100
Collagen I-AF555	Bioss Antibody	Cat# bsm-33400M-A555	1:200
Nucleocapsid CoV-2	Synaptic systems	Cat.No. HS-452 011	1:100
CD3-iFluor790	AAT Bioquest, Inc	100320M0	1:50
PNAd-AF647	Biolegend	Cat.#120807 RRID:AB_2783059	1:50
Pax5-AF647	Biolegend	Cat.#649703 RRID:AB_2562424	1:50
Sytox green	Thermo Fisher	#57020	1:40.000
**Biological samples**			
Human autopsy lung FFPE blocks (COVID and Controls)	Department of Pathology, Charité	https://pathologie-ccm.charite.de/	
Human autopsy lung cryo blocks (COVID)	Department of Neuropathology	https://neuropathologie.charite.de/	
Human autopsy lung cryo blocks (Controls)	NeuroCure BrainBank/Biobank	https://neuropathologie.charite.de/en/research/brainbank/	
Human autopsy lung FFPE blocks (Controls)	NeuroCure BrainBank/Biobank	https://neuropathologie.charite.de/en/research/brainbank/	
Human autopsy lung draining lymph nodes cryo blocks (COVID)	Department of Neuropathology	https://neuropathologie.charite.de/	
Human autopsy lung draining lymph nodes cryo blocks (Controls)	NeuroCure BrainBank/Biobank	https://neuropathologie.charite.de/en/research/brainbank/	
**Chemicals, peptides, and recombinant proteins**			
3-aminopropyltriethoxysilane (APES)	Sigma Aldrich	CAS: 919-30-2	
electron microscopy grade 2% paraformaldehyde	Electron Microscopy Sciences	Cat.No. 50-980-493	
Quadrol solution (N,N,N′,N′-Tetrakis-(2-hydroxypropyl)-ethylendiamin)	Sigma-Aldrich	122262	
CHAPS solution ((3-[3-cholamidopropyl)dimethylammonio]-1-1propanesulfonate	Sigma-Aldrich	226947	
SmartClear Pro	LifeCanvas Technologies		
EasyIndex	Cairn Research	DLC/EI-Z1001	
silica matching liquid	Cargille Laboratories	Cat#19569	

2-Methylbutane 99 +% (GC)	Sigma-Aldrich Chemie AG	M32631	
Pikrin-Fuchsin van Gieson	Th. Geyer Berlin GmbH	2E 050	
Blueing reagent	Roche	05266769001 / 760-2037	
iView DAB,Benchm.Detection Kit	Roche	05266157001 / 760-091	
Deparaffination solution (10x)	Roche	05279771001 / 950-102	
reaction buffer (APK) (10X)	Roche	05353955001 / 950-300	
Haemalaunsolution Mayer		ME 9249	
Glutaraldehyde 25%	SERVA	SVA 23115.01	
iron-Haematoxylin Weig A	Th. Geyer Berlin GmbH	2E 32	
iron-Haematoxylin Weig B	Th. Geyer Berlin GmbH	2E 52	
SHIELD 500ml kit:<br>SHIELD Epoxy (SH-Ex)<br>SHIELD Buffer (SH-BS)<br>SHIELD On (SH-ON)	Cairn Research	DLC/SH-500	
**Commercial assays**			
MagNAPure 96 DNA and Viral NA Large Volume kit	Roche	06374891001	
Rhonda PCR rapid COVID-19 test	Spindiag	SD003-02-020-A01	
Qubit dsDNA HS Assay kit	Thermo Fisher Scientific	Q32854	
10x Visium Spatial Gene Expression Kit	10x Genomics	1000187	
10x Genomics Visium Spatial Tissue Optimization Kit	10x Genomics	1000193	
Chromium Single Cell 3' V3.1 library preparation kit	10x Genomics	PN-1000121	
**Software and algorithms**			
Atlas 5 software	Zeiss	zeiss.com/atlas5	
MelTec TIC-Control 3.0	MelTec GmbH & Co.KG Magdeburg, Germany		
ImageJ/Fiji	Schindelin et al. 2012	https://doi.org/10.1038/nmeth.2019	
Ilastik 1.3.2	Berg et al. 2019	https://doi.org/10.1038/s41592-019-0582-9	
CellProfiler 3.1.8	Carpenter et al. 2006	DOI: 10.1186/gb-2006-7-10-r100	
GraphPad Prism 9.2.0	Graph Pad Software	www.graphpad.com	
R (version 4.1.0)	R Core Team (2020)	https://www.R-project.org/	

Seurat (version 4.0.4)	https://CRAN.R-project.org/package=Seurat	doi: 10.1038/nbt.3192	
R (version 3.6.1)	R Core Team (2020)	https://www.R-project.org/	
Seurat (version 3.1.1)	https://CRAN.R-project.org/package=Seurat	doi: 10.1038/nbt.3192	
Loupe Browser 5.1.0 software	10x Genomics	https://support.10xgenomics.com/single-cell-gene-expression/software/downloads/latest	
Space Ranger software 1.3.0	10x genomics	https://support.10xgenomics.com/spatial-gene-expression/software/pipelines/latest/installation	
Gene Set Enrichment Analysis (GSEA) *fgsea* package (version 1.18.0)	Molecular Signatures Database v7.4	Subramanian, Tamayo, et al. (2005, PNAS) and Mootha, Lindgren, et al. (2003, Nature Genetics).	
single sample gene set enrichment analysis (ssGSEA) with the *escape* package (version 1.2.0)	Molecular Signatures Database v7.4	Subramanian, Tamayo, et al. (2005, PNAS) and Mootha, Lindgren, et al. (2003, Nature Genetics).	
MSigDB (v7.4)	Molecular Signatures Database v7.4	Subramanian, Tamayo, et al. (2005, PNAS) and Mootha, Lindgren, et al. (2003, Nature Genetics).	
Cell Ranger Software Suite (Version 3.1.0)	10x genomics	https://support.10xgenomics.com/single-cell-gene-expression/software/pipelines/latest/installation	
ImSpector software Version 7.0.73	LaVision Biotec	LaVision BioTec GmbH https://www.lavisionbiotec.com/	
Imaris Bitplanex64 Version 9.7.2.	Andor Technology Ltd.	Andor Technology Ltd. https://imaris.oxinst.com/	
Imaris Stitcher Version 9.7.2.	Andor Technology Ltd.	Andor Technology Ltd. https://imaris.oxinst.com/	
Imaris Converter Version 9.7.2.	Andor Technology Ltd.	Andor Technology Ltd. https://imaris.oxinst.com/	
**Other**			
Toponome Image Cycler® MM3	MelTec GmbH & Co.KG Magdeburg, Germany		
Superfrost Plus Gold slides	Fisher Scientific, Jena, Germany	11976299	
Zeiss LSM 880	Zeiss		
MH560 cryotome	ThermoFisher		
cover slides 24 x 60 mm	Menzel-Gläser	10672-015	

“press-to-seal” silicone sheets	Life technologies	P24745	
Illumina NextSeq 500/550	Illumina		
Nextseq 500	Illumina		
Novaseq 6000 S1	Illumina		
Makro fluoreszenz-cuvette	Msscientific Chromatographie-Handel GmbH, Berlin, Germany	FF-1FL-G-5	
Ultramicroscope UM-0108	LaVision BioTec		
LifeCanvas SmartClear II Pro	Cairn Research, Faversham, United Kingdom	https://lifecanvastech.com/products/smartclear-ii-pro/	
Cork plates, 20mm	Slee medical GmbH	#30001001	
Glas slides, 76 x 26, SuperFrost Plus	Langenbrink	#041300	
Embedding media Paraplast Plus	Roth	X881.1	
OCT Compound, Tissue Tek	Charité	AK 4583 SS	

Supplementary Table 1. **Reagents and Resources**
