# Supplementary material for: Distinct tissue niches direct lung immunopathology via CCL18 and CCL21 in severe COVID-19
Source: Nat Commun. 2023 Feb 11;14:791. doi: 10.1038/s41467-023-36333-2 (PMC9922044; doi:10.1038/s41467-023-36333-2)
Supplement: Supplementary file 3 — Description of Additional Supplementary Files [file 41467_2023_36333_MOESM3_ESM.pdf]

Description of Additional Supplementary Files:

Supplementary Movie 1. LSFM of COVID-19 lung tissue from acute case 3 stained with antibodies against ER-TR7 (yellow) for visualization of the fibroblast and reticular fiber network.

Supplementary Movie 2. LSFM of COVID-19 lung tissue from prolonged case 5 stained with anti-ER-TR7 (yellow) for visualization of the fibroblast and reticular fiber network.

Supplementary Movie 3. LSFM of COVID-19 lung from acute case 3 showing a thrombus attached to the vessel wall. Yellow color represents ER-TR7 staining, signals shown in magenta result from autofluorescence.

Supplementary Movie 4. LSFM of COVID-19 lung from acute case 3 stained with SYTOX green (nuclei) in magenta and anti-CD3 in cyan.

Supplementary Movie 5. LSFM of COVID-19 lung from prolonged case 5 stained with SYTOX green (nuclei) in magenta and anti-CD3 in cyan.
