# Supplementary material for: Distinct tissue niches direct lung immunopathology via CCL18 and CCL21 in severe COVID-19
Source: Nat Commun. 2023 Feb 11;14:791. doi: 10.1038/s41467-023-36333-2 (PMC9922044; doi:10.1038/s41467-023-36333-2)
Supplement: Supplementary file 9 — Reporting Summary [file 41467_2023_36333_MOESM9_ESM.pdf]

nature portfolio

Corresponding author(s): Anja Hauser

Last updated by author(s): Dec 21, 2022

# Reporting Summary

Nature Portfolio wishes to improve the reproducibility of the work that we publish. This form provides structure for consistency and transparency in reporting. For further information on Nature Portfolio policies, see our Editorial Policies and the Editorial Policy Checklist.

## Statistics

For all statistical analyses, confirm that the following items are present in the figure legend, table legend, main text, or Methods section.

| n/a | Confirmed | |
|---|---|---|
| ☐ | ☒ | The exact sample size (*n*) for each experimental group/condition, given as a discrete number and unit of measurement |
| ☐ | ☒ | A statement on whether measurements were taken from distinct samples or whether the same sample was measured repeatedly |
| ☐ | ☒ | The statistical test(s) used AND whether they are one- or two-sided<br>*Only common tests should be described solely by name; describe more complex techniques in the Methods section.* |
| ☒ | ☐ | A description of all covariates tested |
| ☐ | ☒ | A description of any assumptions or corrections, such as tests of normality and adjustment for multiple comparisons |
| ☐ | ☒ | A full description of the statistical parameters including central tendency (e.g. means) or other basic estimates (e.g. regression coefficient) AND variation (e.g. standard deviation) or associated estimates of uncertainty (e.g. confidence intervals) |
| ☐ | ☒ | For null hypothesis testing, the test statistic (e.g. *F*, *t*, *r*) with confidence intervals, effect sizes, degrees of freedom and *P* value noted<br>*Give P values as exact values whenever suitable.* |
| ☒ | ☐ | For Bayesian analysis, information on the choice of priors and Markov chain Monte Carlo settings |
| ☒ | ☐ | For hierarchical and complex designs, identification of the appropriate level for tests and full reporting of outcomes |
| ☐ | ☒ | Estimates of effect sizes (e.g. Cohen's *d*, Pearson's *r*), indicating how they were calculated |

*Our web collection on statistics for biologists contains articles on many of the points above.*

## Software and code

Policy information about availability of computer code

| | |
|---|---|
| Data collection | The software TIC-Control (MelTec) version 3.0 was used to control the pipetting robot and the microscope system used for multiplexed histology data acquisition and ImSpector software Version 7.0.73 was used for LSFM data acquisition |
| Data analysis | ImageJ 1.52i was used to pre-process and normalize fluorescence images.<br>Ilastik 1.3.2 was used to perform pixel classification<br>CellProfiler 3.1.8 was used for cell segmentation, for extracting single-cell features (mean fluorescence intensities per cell)<br>GraphPad Prism 9.2.0 was use for statistics<br>R version 4.1.0 was used for ST and multiplexed histology data analysis and version 3.6.1 for snRNA seq data analysis<br>Seurat version 4.0.4 was used for ST and multiplexed histology data analysis and version 3.1.1 for snRNA seq data analysis<br>Loupe Browser 5.1.0 software was used for ST data analysis<br>Space Ranger software 1.3.0 was used for ST data analysis<br>Gene Set Enrichment Analysis (GSEA) fgsea package (version 1.18.0),<br>single sample gene set enrichment analysis (ssGSEA) with the escape package (version 1.2.0) and<br>MSigDB (v7.4) was used for gene data analysis<br>Imaris Bitplanex64 Version 9.7.2, Imaris Stitcher Version 9.7.2 and Imaris Converter Version 9.7.2 were used for LSFM data processing<br><br>All software used for data acquisition and data analysis has already been published elsewhere and no new code has been written. |

For manuscripts utilizing custom algorithms or software that are central to the research but not yet described in published literature, software must be made available to editors and reviewers. We strongly encourage code deposition in a community repository (e.g. GitHub). See the Nature Portfolio guidelines for submitting code & software for further information.

## Data

Policy information about availability of data

All manuscripts must include a data availability statement. This statement should provide the following information, where applicable:

- Accession codes, unique identifiers, or web links for publicly available datasets
- A description of any restrictions on data availability
- For clinical datasets or third party data, please ensure that the statement adheres to our policy

De-identified human/patient spatial transcriptomics data have been deposited at Gene Expression Omnibus (https://www.ncbi.nlm.nih.gov/geo/), under record GSE190732. To review GEO accession GSE190732, go to https://www.ncbi.nlm.nih.gov/geo/query/acc.cgi?acc=GSE190732. Multiplexed histology data, as well as feature matrices and spatial coordinates required to re-analyze the data reported in this study are publicly available in the Zenodo repository under DOI: 10.5281/zenodo.7447490 and all linked / related identifiers. All other microscopy data, as well as any additional information required to re-analyze the data reported in this paper will be shared by the lead contact upon request. No original code was used in this study.

# Field-specific reporting

Please select the one below that is the best fit for your research. If you are not sure, read the appropriate sections before making your selection.

☒ Life sciences ☐ Behavioural & social sciences ☐ Ecological, evolutionary & environmental sciences

For a reference copy of the document with all sections, see nature.com/documents/nr-reporting-summary-flat.pdf

# Life sciences study design

All studies must disclose on these points even when the disclosure is negative.

| | |
|---|---|
| Sample size | During the period of 03/2020 and 09/2020, all possible prolonged COVID-19 donors receiving an autopsy at Charité-Universitätsmedizin Berlin, where COVID-19 diagnosis was given and respective tissue was available for analysis, were included in this study. Randomly (age and sex matched) acute, chronic and control cases were selected among the cases with available tissue. We analyzed postmortal tissue samples from 17 human donors (4 control, 3 acute cases of COVID-19 disease, 5 cases of chronic COVID-19 disease and 5 cases of prolonged COVID-19 disease). No statistical method was used to predetermine sample size. |
| Data exclusions | All donors with a pulmonary tumor, hematologic stem cell therapy or autoimmune comorbidity were excluded. We used the same exclusion criteria for controls, but with negative PCR and immunohistochemistry for SARS-CoV-2, as additional inclusion criteria.<br>One control case analyzed by multiplexed histology had to be excluded due to postmortem PCR and immunohistochemical prove of a clinical unremarkable SARS-CoV-2 infection.<br>Two out of fourteen tissue sections analyzed by ST gave very low cDNA amounts. Although these were used for library preparation, they did not pass the quality control afterwards. These two tissue sections and the data extracted from them were not used for further analysis or for manuscript preparation. |
| Replication | At least 2 different areas from each sample have been analyzed by multiplexed microscopy. Experiments have been performed in a minimum of 3 replicates per disease group. All attempts at replication were successful, except for two out of fourteen tissue sections analyzed by ST, which gave very low cDNA amounts. They were nevertheless used for library preparation, but did not pass the quality control afterwards. These two tissue sections and the data extracted from them were not used for further analysis or for manuscript preparation. |
| Randomization | Randomization is not relevant to our study, as sample stratification was performed based on disease duration to interrogate differences based on that particular clinical parameter. |
| Blinding | Evaluation of histological and immunohistochemical staining (tissue annotations and fibrosis scoring) was performed separately by at least two independent raters that were blinded for disease duration. Multiplexed histology, electron microscopy, light sheet fluorescence microscopy, snRNAseq and ST data acquisition, processing and analysis were performed unblinded. |

# Reporting for specific materials, systems and methods

We require information from authors about some types of materials, experimental systems and methods used in many studies. Here, indicate whether each material, system or method listed is relevant to your study. If you are not sure if a list item applies to your research, read the appropriate section before selecting a response.

## Materials & experimental systems

| n/a | Involved in the study |
|---|---|
| ☐ | ☒ Antibodies |
| ☒ | ☐ Eukaryotic cell lines |
| ☒ | ☐ Palaeontology and archaeology |
| ☒ | ☐ Animals and other organisms |
| ☐ | ☒ Human research participants |
| ☒ | ☐ Clinical data |
| ☒ | ☐ Dual use research of concern |

## Methods

| n/a | Involved in the study |
|---|---|
| ☒ | ☐ ChIP-seq |
| ☒ | ☐ Flow cytometry |
| ☒ | ☐ MRI-based neuroimaging |

# Antibodies

Antibodies used

Fibronectin; Thermo Fisher Cat# PA5-29578; RRID:AB_2547054, Dilution 1:200
Rabbit IgG-PE; Rockland Cat# 711-708-127, RRID:AB_218957, Dilution 1:200
CCR2-PE; Miltenyi Biotec Cat# 130-118-338, RRID:AB_2751486, Dilution 1:50
CD163-PE; Biolegend Cat# 333605, RRID:AB_1134005, Dilution 1:50
CD56-PE; Miltenyi Biotec Cat# 130-098-137, RRID:AB_2661200, Dilution 1:50
CCR8-APC; BioLegend Cat# 360609, RRID:AB_2820017, Dilution 1:50
CD1c-PE; Miltenyi Biotec Cat# 130-113-864, RRID:AB_2726358, Dilution 1:50
CD3-PE; Miltenyi Biotec Cat# 130-113-139, RRID:AB_2725967, Dilution 1:50
CD14-PE; Miltenyi Biotec Cat# 130-113-709, RRID:AB_2726250, Dilution 1:50
CCR7-PE; Miltenyi Biotec Cat# 130-120-603, RRID:AB_2784046, Dilution 1:50
Eomes-PE; Thermo Fisher Cat# 14-4877-80, RRID:AB_2572881, Dilution 1:50
CD45-PE; Miltenyi Biotec Cat# 130-113-118, RRID:AB_2725946, Dilution 1:50
CXCR3-PE; Miltenyi Biotec Cat# 130-101-379, RRID:AB_2655734, Dilution 1:10
PD1-PE; Miltenyi Biotec Cat# 130-120-388, RRID:AB_2752074, Dilution 1:50
CD16-PE; Miltenyi Biotec Cat# 130-113-955, RRID:AB_2726428, Dilution 1:50
CD93-PE; Miltenyi Biotec Cat# 130-098-436, RRID:AB_2659615, Dilution 1:10
CD4-PE; Miltenyi Biotec Cat# 130-113-214, RRID:AB_2726025, Dilution 1:50
Granzyme A-PE; Miltenyi Biotec Cat# 130-123-973, RRID:AB_2889678, Dilution 1:10
CD31-PE; R and D Systems Cat# FAB3567P, RRID:AB_2279388, Dilution 1:50
ICOS-PE; Miltenyi Biotec Cat# 130-120-155, RRID:AB_2784102, Dilution 1:50
TREM1-PE; Miltenyi Biotec Cat# 130-101-033, RRID:AB_2657706, Dilution 1:10
CD20-PE; Miltenyi Biotec Cat# 130-113-374, RRID:AB_2726143, Dilution 1:50
CD11b-PE; Miltenyi Biotec Cat# 130-110-553, RRID:AB_2654665, Dilution 1:50
CD8-PE; Miltenyi Biotec Cat# 130-113-720, RRID:AB_2726261, Dilution 1:50
CD68-PE; Miltenyi Biotec Cat# 130-118-486, RRID:AB_2784270, Dilution 1:50
CD127-PE; Miltenyi Biotec Cat# 130-113-414, RRID:AB_2733759, Dilution 1:50
CD11c-PE; Miltenyi Biotec Cat# 130-113-580, RRID:AB_2726180, Dilution 1:50
Granzyme B-PE; Miltenyi Biotec Cat# 130-116-654, RRID:AB_2727639, Dilution 1:50
CD69-PE; Miltenyi Biotec Cat# 130-112-613, RRID:AB_2659065, Dilution 1:50
CD94-PE; Miltenyi Biotec Cat# 130-098-973, RRID:AB_2659624, Dilution 1:10
CD141-PE; Miltenyi Biotec Cat# 130-114-188, RRID:AB_2751233, Dilution 1:50
CD27-PE; Miltenyi Biotec Cat# 130-114-166, RRID:AB_2726471, Dilution 1:50
CD38-PE; Miltenyi Biotec Cat# 130-113-427, RRID:AB_2733813, Dilution 1:50
CD57-PE; Miltenyi Biotec Cat# 130-111-963, RRID:AB_2658747, Dilution 1:50
CD161-PE; Miltenyi Biotec Cat# 130-114-119, RRID:AB_2733771, Dilution 1:50
HLA-DR,DP,DQ-PE; Miltenyi Biotec Cat# 130-120-715, RRID:AB_2752176, Dilution 1:50
CD34-PE; Miltenyi Biotec Cat# 130-113-741, RRID:AB_2726281, Dilution 1:50
CD66b-PE; Miltenyi Biotec Cat# 130-122-966, RRID:AB_2811418, Dilution 1:50
Pancytokeratin-PE; Arigo Biolaboratories Cat# ARG56130, Dilution 1:50
Ki67-FITC; Dako F268, Dilution 1:50
CD49a-PE; Miltenyi Biotec Cat# 328304, RRID:AB_1236407, Dilution 1:50
Collagen IV-FITC; Antibodies-Online Cat# ABIN376119, RRID:AB_10763557, Dilution 1:500
ER-TR7-PE; Thermo Fisher Cat# MA1-40076, RRID:AB_1074409, Dilution 1:200
SMA-FITC; Abcam Cat# ab8211, RRID:AB_306359, Dilution 1:100
CD45RA-PE; Miltenyi Biotec Cat# 130-113-366, RRID:AB_2726136, Dilution 1:50
C1q-FITC; DAKO Cat# F0254, RRID:AB_2335713, Dilution 1:50
MRP14-PE; Miltenyi Biotec Cat# 130-114-516, RRID:AB_2726684, Dilution 1:50
Collagen I-PE; Biolegend Cat# 303126, RRID:AB_2563303, Dilution 1:50
CD45-AF647; Santa Cruz Biotechnology Cat# sc-1178, RRID:AB_627074, Dilution 1:50
CD3-AF647; Biolegend Cat# 344825, RRID:AB_2563440, Dilution 1:50
CD163-AF647; Biolegend Cat# 326508, RRID:AB_893264, Dilution 1:50
ER-TR7-AF546; Santa Cruz Biotechnology Cat# sc-73355, RRID:AB_1122890, Dilution 1:100

| | |
|---|---|
| | Collagen I-AF555; Bioss Antibody Cat# bsm-33400M-A555, Dilution 1:100<br>Nucleocapsid CoV-2; Synaptic systems Cat.No. HS-452 011, Dilution 1:4000<br>CD3-iFluor790; AAT Bioquest, Inc 100320M0, Dilution 1:50<br>Pax5-AF647; Biolegend Cat.#649703, RRID:AB_2562424, Dilution 1:50<br>PNAd-AF647; Biolegend Cat.#120807, RRID:AB_2783059, Dilution 1:50<br>Sytox green; Thermo Fisher #57020, Dilution 1:40000<br>DAPI; Roche Cat# 10236276001, Dilution 1:5000<br>IgA-PE; Miltenyi Biotec Cat# 130-114-002; RRID:AB_2733860, Dilution 1:50<br>IgA2-PE; Miltenyi Biotec Cat# 130-117-874; RRID:AB_2728061, Dilution 1:50<br>IgM-PE; Miltenyi Biotec Cat# 130-122-930; RRID:AB_2801972, Dilution 1:50<br>IgG-PE; Miltenyi Biotec Cat# 130-119-964; RRID:AB_2751950, Dilution 1:50 |
| Validation | All antibodies are commercially available and have been validated for flow cytometry and/or immunofluorescence by the manufacturers. We have titrated all antibodies for immunofluorescence and tested them all in several human tissues (including skin, thymus, SI, colon, kidney, LN, liver, cervix, tonsil and lung) assessing by visual inspection the pattern of expression in the tissues, expression level, sub-cellular distribution and co-localization with other lineage-defining markers. |

## Human research participants

Policy information about studies involving human research participants

| | |
|---|---|
| Population characteristics | This study was conducted using post-mortem tissues from 17 human donors, who passed away during the COVID-19 pandemic in Berlin. Mean age was 67,8 years (range 56 to 94 years), included were 3 females and 14 men. Biological sex was assigned during autopsy according to the presence of the respective sex organs and reported as female or male. Gender was not considered due to the postmortem study cohort design. We did not perform sex-based analyses, since the very low sample size, in particular for females, would not allow for meaningful conclusions. Data is reported disaggregated for sex in Table 1 and in the source data files. Medical side conditions varied between individuals, all clinical details are listed in Table 1. |
| Recruitment | The lung and lymph node samples included in this study have been collected in the Department of Pathology of Charité - Universitätsmedizin Berlin as part of the COVID-19 autopsy Biobank. |
| Ethics oversight | This study was conducted in accordance with the declaration of Helsinki and with the approval of the Ethics Committee of the Charité (EA 1/144/927 13, EA2/066/20 and EA1/317/20) and the Charité - BIH COVID-19 research board. Autopsies were performed on the legal basis of §1 926 SRegG BE of the autopsy act of Berlin and §25(4) of the German Infection Protection Act. Autopsy consent was obtained from the families of the patients. |

Note that full information on the approval of the study protocol must also be provided in the manuscript.
